# Supplementary material for: Mechanical stress determines the configuration of TGFβ activation in articular cartilage
Source: Nat Commun. 2021 Mar 17;12:1706. doi: 10.1038/s41467-021-21948-0 (PMC7969741; doi:10.1038/s41467-021-21948-0)
Supplement: Supplementary file 1 — Supplementary Information [file 41467_2021_21948_MOESM1_ESM.pdf]

SFig. 1

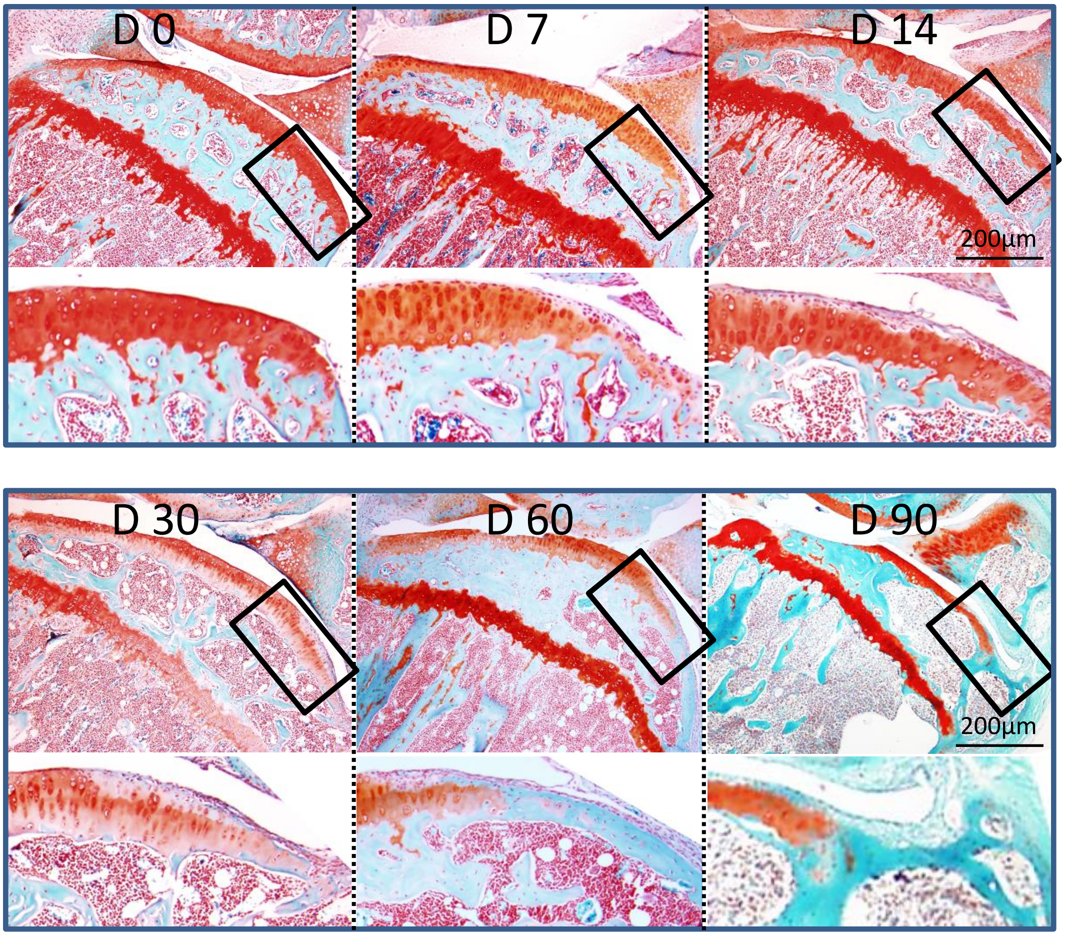

Supplementary Fig. 1 Cartilage degeneration starts from anterior region of tibia medial plateau after ACLT. Safranin O-fast green staining of sagittal sections of proximal tibia of mice that sacrificed at 0, 7, 14, 30, and 60 days post ACLT surgery. The earliest sign of proteoglycan loss and cartilage fibrillation as indicated by discoloration of safranin O staining were observed at the anterior region (boxed) and then gradually extended posteriorly as time pass by. Osteophytes formation also primarily formed at anterior region. n=5 biological independent animals.

SFig. 2

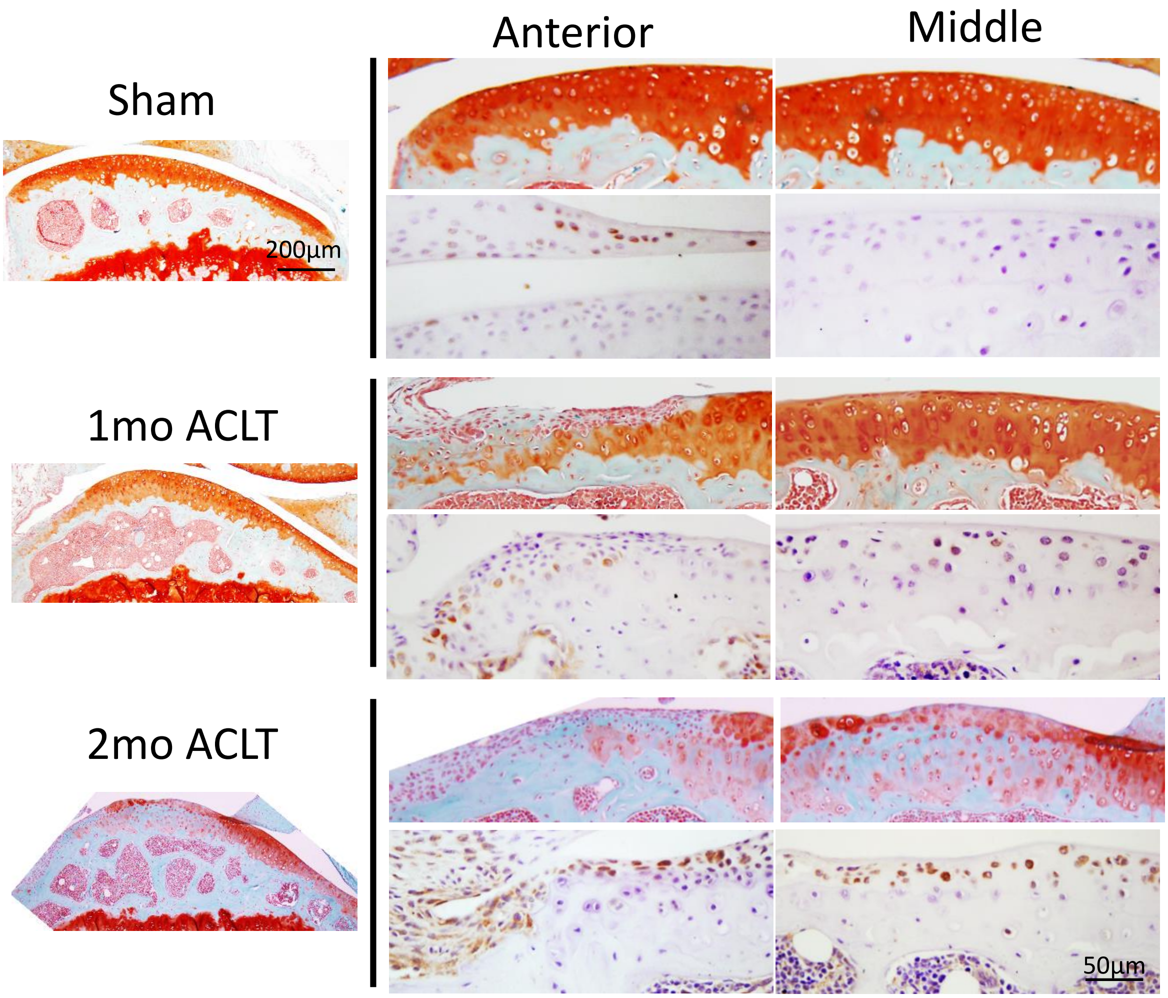

Supplementary Fig. 2 The majority of αV integrin positive cells were found localized at the cartilage with proteoglycan loss after ACLT. Mice received sham operation or ACLT were sacrificed at 1 month or 2 months post surgery. Left panel is the Safranin O-fast green staining of sagittal sections of proximal tibia. Right panels are the magnified images of the anterior or middle region of left panel. Upper rows are Safranin O-fast green staining and lower rows are immunohistochemistry staining of αVβ6 (brown). The αVβ6 positive cells were barely observed in sham operated mice cartilage whereas remarkably increased in ACLT mice and mainly are localized in the area with proteoglycan loss. n=8 biological independent animals.

SFig. 3

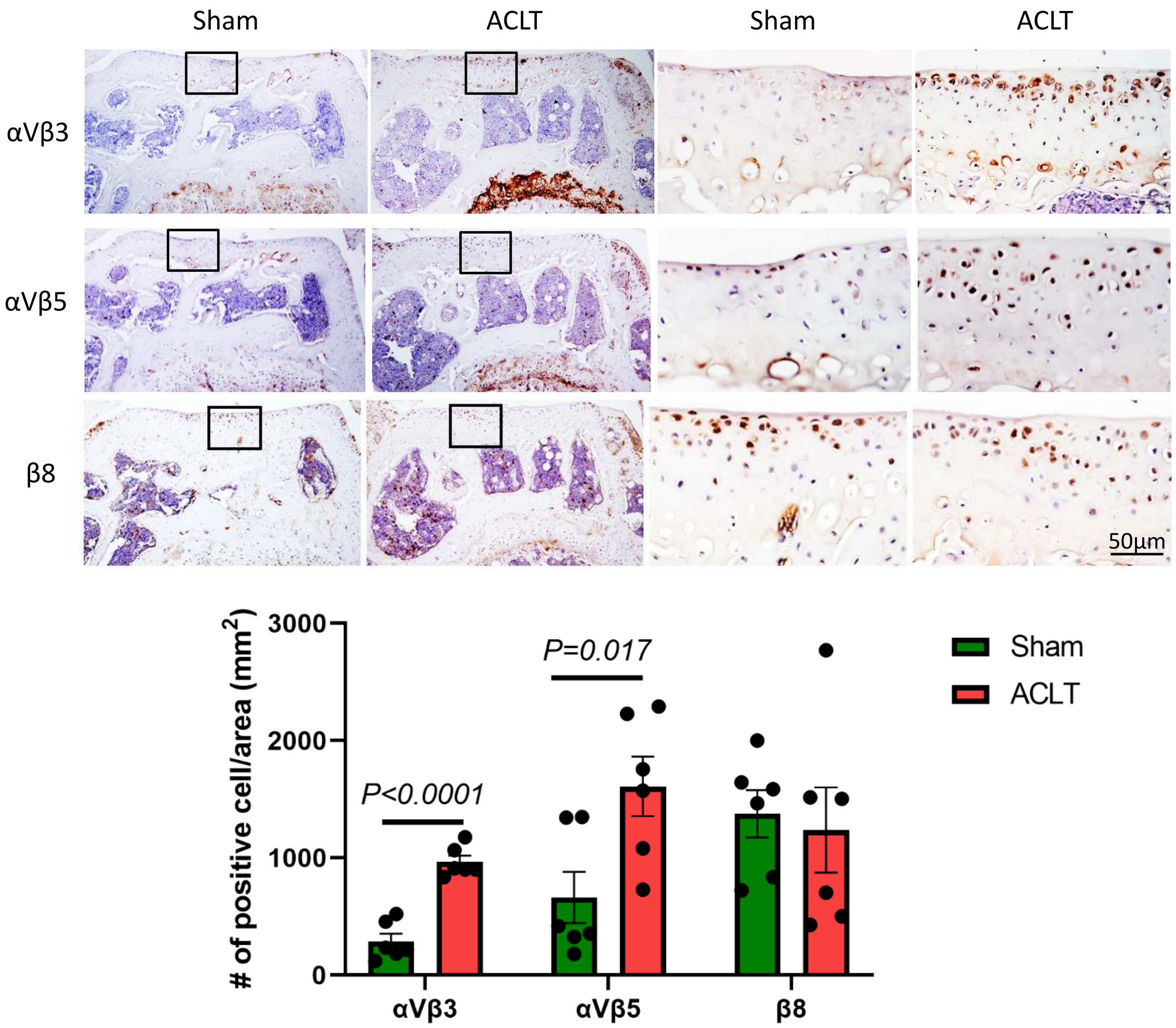

Supplementary Fig. 3 The expression of other αV subunit containing integrins were also increased in AC post ACLT. Mice received sham operation or ACLT were sacrificed at 1-month post surgery. Immunohistochemistry staining of αVβ3, αVβ5 and β3 was performed in coronal sections of medial compartment of tibiae plateau. The positive cells were labeled brown. The number of αVβ3 and αVβ5 cells was remarkably elevated in the AC of ACLT mice as compared to that of sham operated mice. n=6 biologically independent animals. Data are presented as mean values +/- SEM. Data was analyzed using two tail unpaired t-test.

SFig. 4

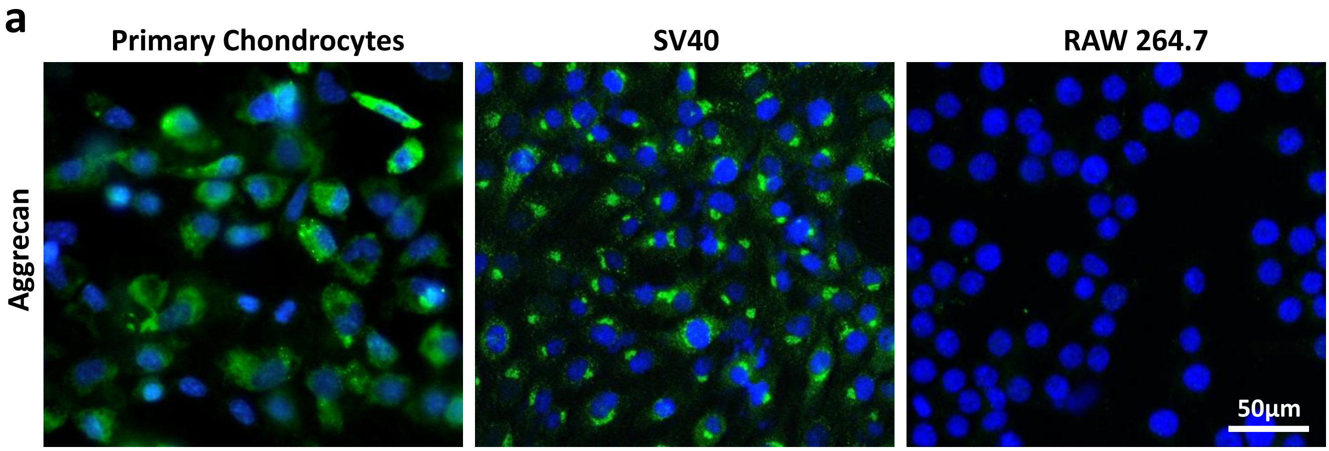

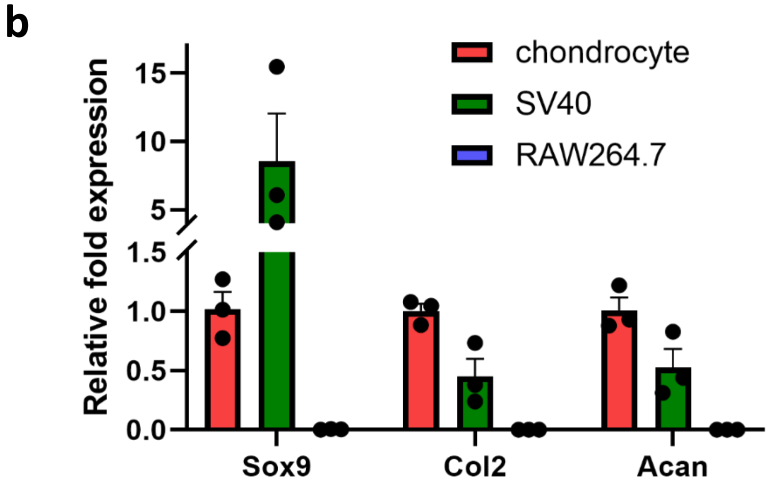

Supplementary Fig. 4 Characterization of the SV40 cell line. Primary chondrocytes were isolated from the knee joint of the neonatal at day 7. RAW 264.7 is the pre-osteoclast cell line that is known to be negative for the selected chondrocyte markers. (**a**) Immunofluorescence staining of aggrecan (green). (**b**) Q-PCR of Sox9, type II collagen (Col2) and aggrecan (Acan) in primary chondrocytes, SV40, and RAW 264.7 cells. n=3 independent experiments. Data are presented as mean values +/- SEM.

SFig. 5

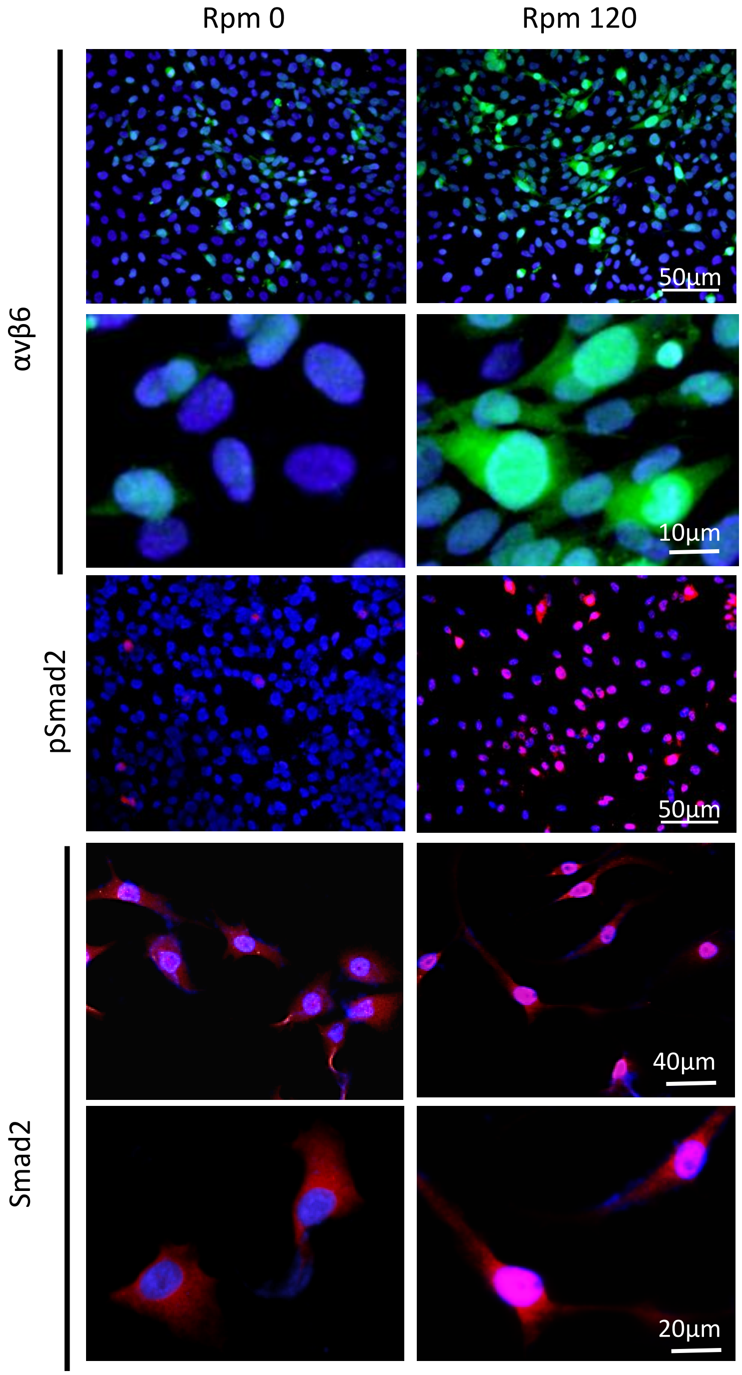

Supplementary Fig. 5 Mechanical stress increased the expression of αVβ6 and TGFβ activity in chondrocytes. SV40 immortalized chondrocyte cell lines were subjected to shear stress at 0 dyne/cm^2 or 6.58 dynes/cm^2 for 48 hours. Immunofluorescence staining of αVβ6, pSmad2 and Smad2 in the cells with or without shear stress. Elevated αVβ6 expression, Smad2 phosphorylation and nuclear translocation were observed in cells that subjected to shear stress. Experiments were repeated 3 times independently.

SFig. 6

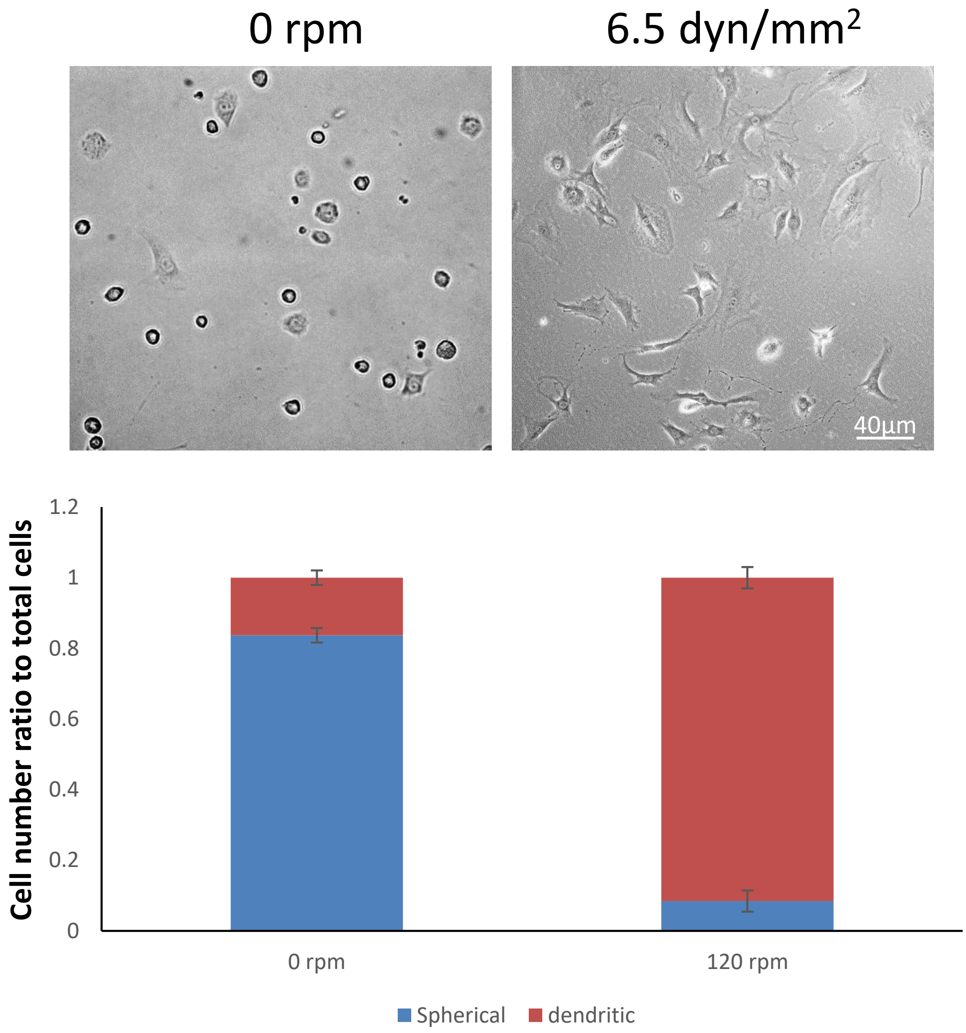

Supplementary Fig. 6 Shear stress changed the morphology of primary chondrocytes. Primary chondrocytes that isolated from neonatal mice were subjected to shear stress at 0 dyne/cm^2 or 6.58 dynes/cm^2 for 24 hours. The phase contrast images were taken under inverted microscope. The majority of the cells that cultured in free well were in a spherical shape whereas the cells changed their morphology to dendritic/spindle like shape after challenged by shear stress. The n=3 independent experiments. Data are presented as mean values +/- SEM.

SFig. 7

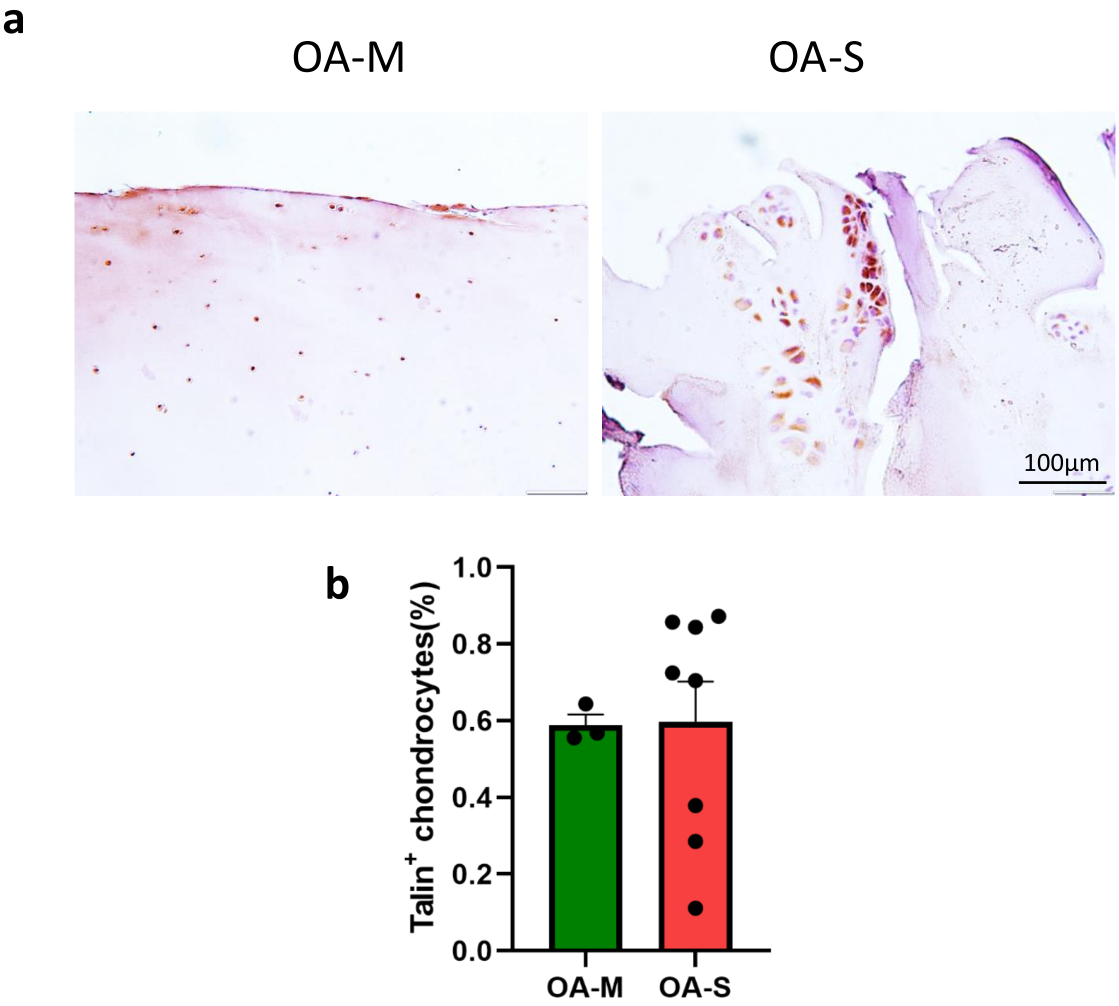

Supplementary Fig. 7 Talin is expressed by chondrocytes in human OA cartilage. (a) Immunohistological staining of talin in the articular cartilage of human tibia plateau. (**b**) Quantitative analysis of the percentage of talin positive chondrocytes in the articular cartilage. The intensity of talin is slightly higher in the clustered hypertrophic chondrocytes in OA-S specimens but the percentage of the talin$^+$ cells are comparable in the two groups. OA-M: OA specimen with minimal cartilage degeneration, OA-S: OA specimen with severe cartilage degeneration. n=3 or 8 biologically independent specimens in OA-M or OA-S groups, respectively. Data are presented as mean values +/- SEM.

SFig. 8

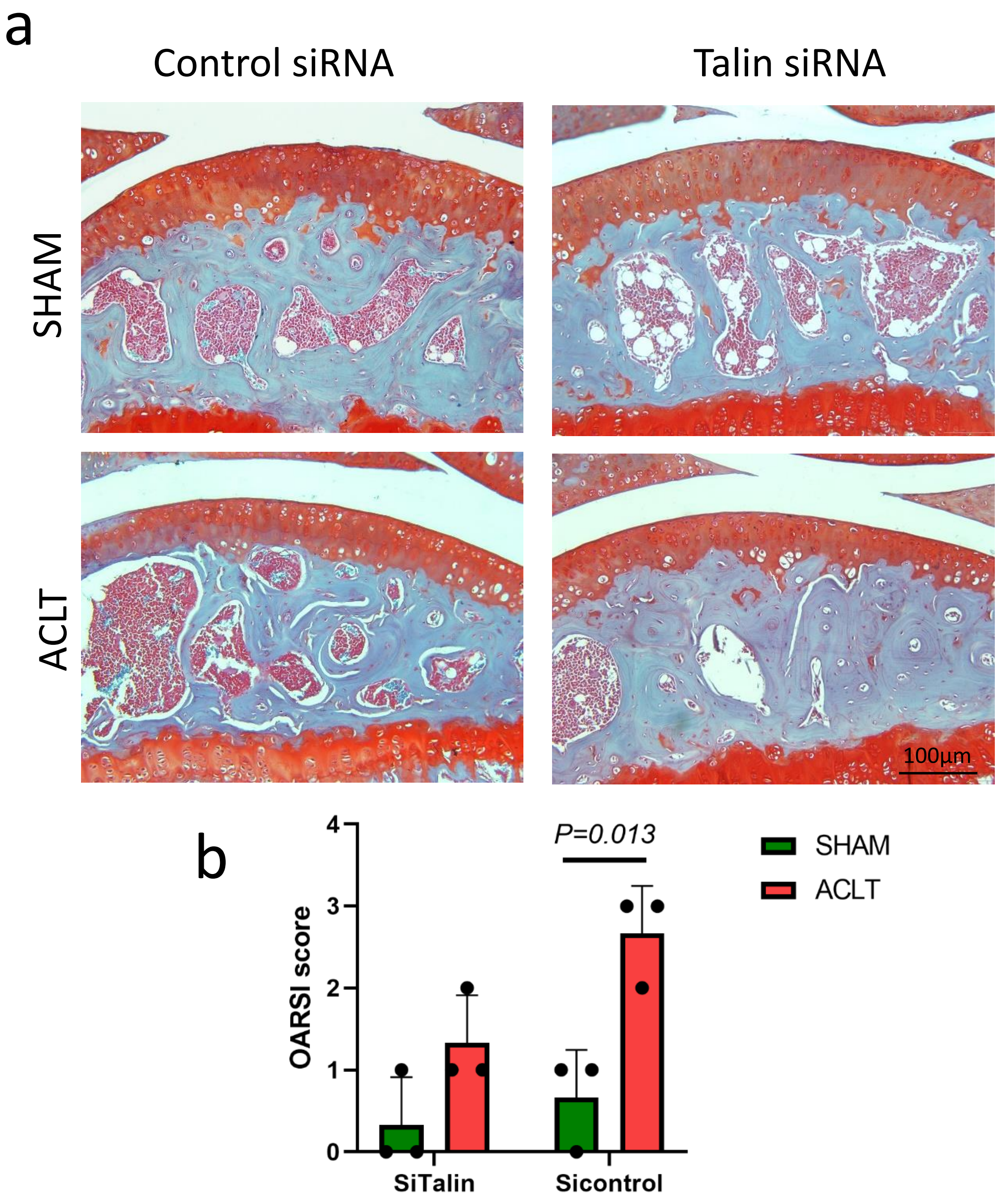

Supplementary Fig. 8 Knockdown the expression of Talin in chondrocytes prevented cartilage degeneration in OA mouse model. (a) Representative image of safranin O fast green staining of AC of knee joints that harvested from C57BL/6 mice that subjected to and intra-articular injection of si-Talin or control siRNA for one-month post ACLT or sham surgery. (b) OARSI score analysis. n=4 biologically independent animals. Data are presented as mean values +/- SEM. Data was analyzed using two tailed unpaired t-test.

SFig. 9

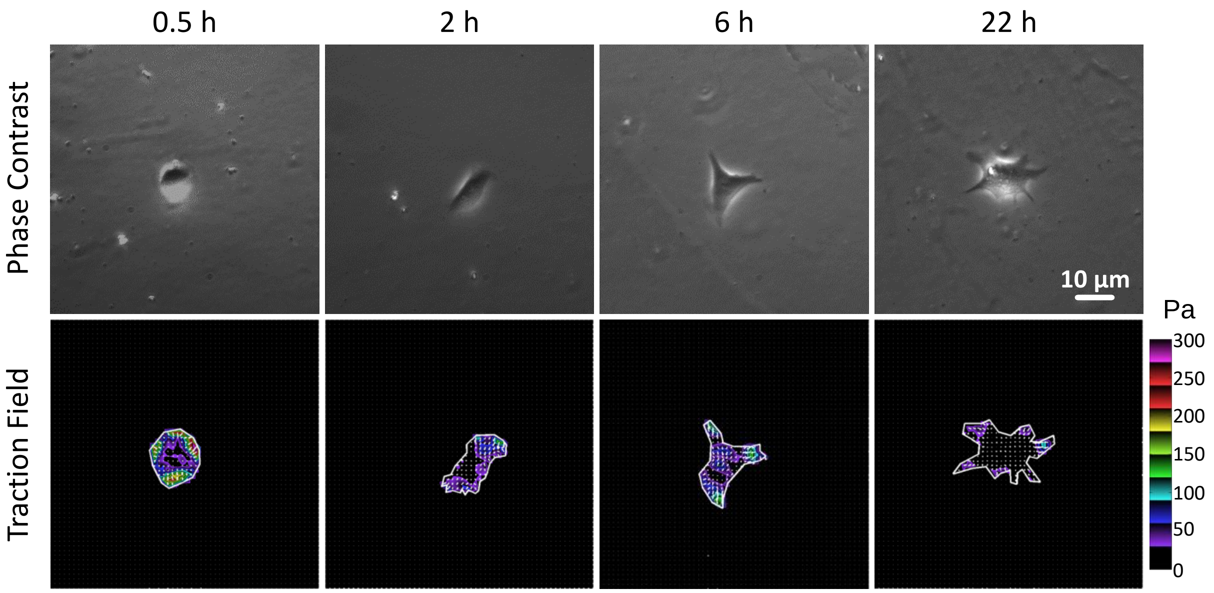

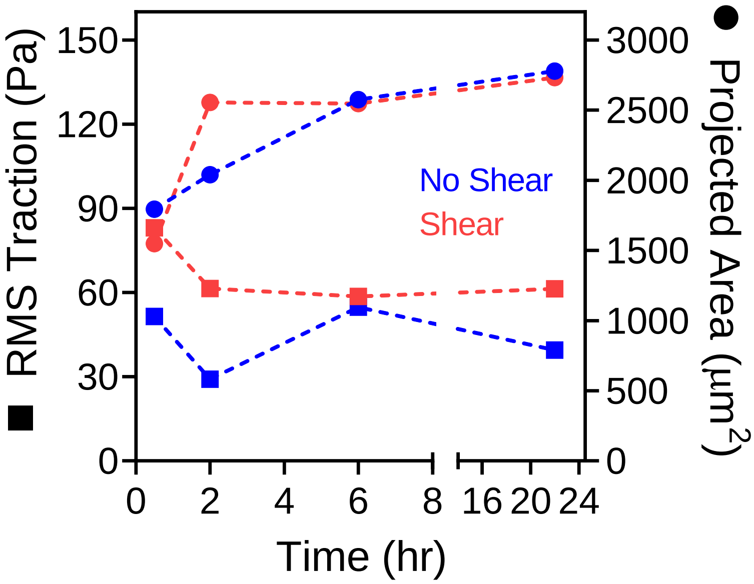

Supplementary Fig. 9 Time course of cell traction forces during cell adhesion. SV40 immortalized chondrocyte cell lines were subjected to shear stress at 0 dyne/cm^2 or 6.58 dynes/cm^2 for 48 hours. Then the cells were plated on the inert elastic polyacrylamide gel blocks and allowed to adhere for different time course. The contractile force that generated by the cells and cell projected areas were plotted. The maximum difference between shear and no shear group were observed at 2 hours post adherent. The experiments were repeated 3 times independently.

SFig. 10

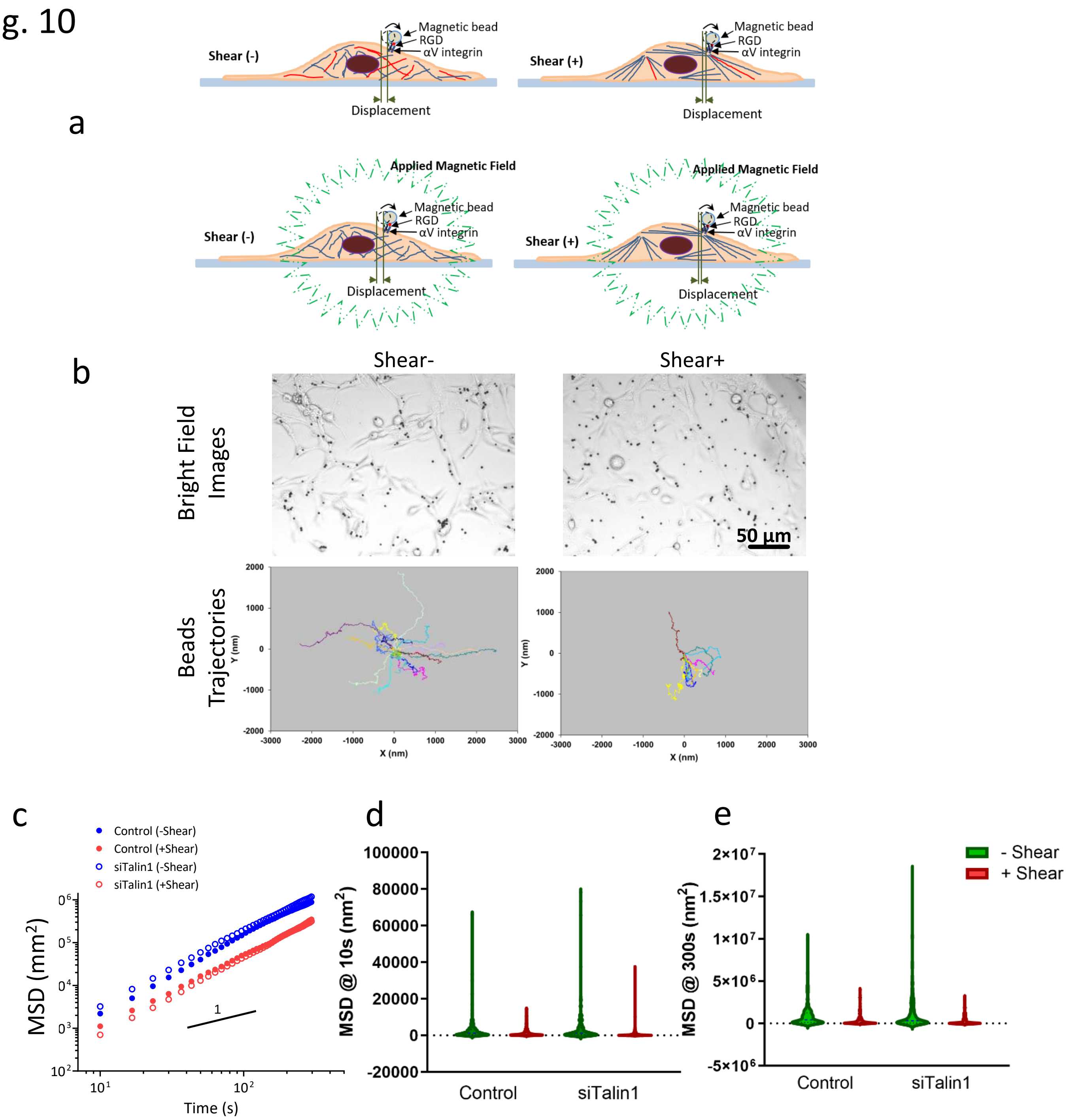

Supplementary Fig. 10 Spontaneous bead motions is not dependent on Talin1. (A) Schematic illustration of the displacement of magnetic beads with or with out magnetic force. (**B-E**) RGD-coated ferrimagnetic beads labeled SV40 immortalized chondrocyte cells were subjected to shear stress at 0 dyne/cm^2 or 6.58 dynes/cm^2 for 48 hours. n=174-258 independent cells. Mean has been shown in dotted line. The spontaneous nanoscale movements of individual beads bound to adherent cells were recorded and characterized by computing the mean square displacement of the beads as a function of time [MSD(t)] (nm^2). We found that the spontaneous movement of the beads was significantly reduced in shear group as compared to that of controls, which indicated that the cytoskeleton remodeling was reduced in cells that subjected to mechanical stress and this process was independent of the action of Talin1.

SFig. 11

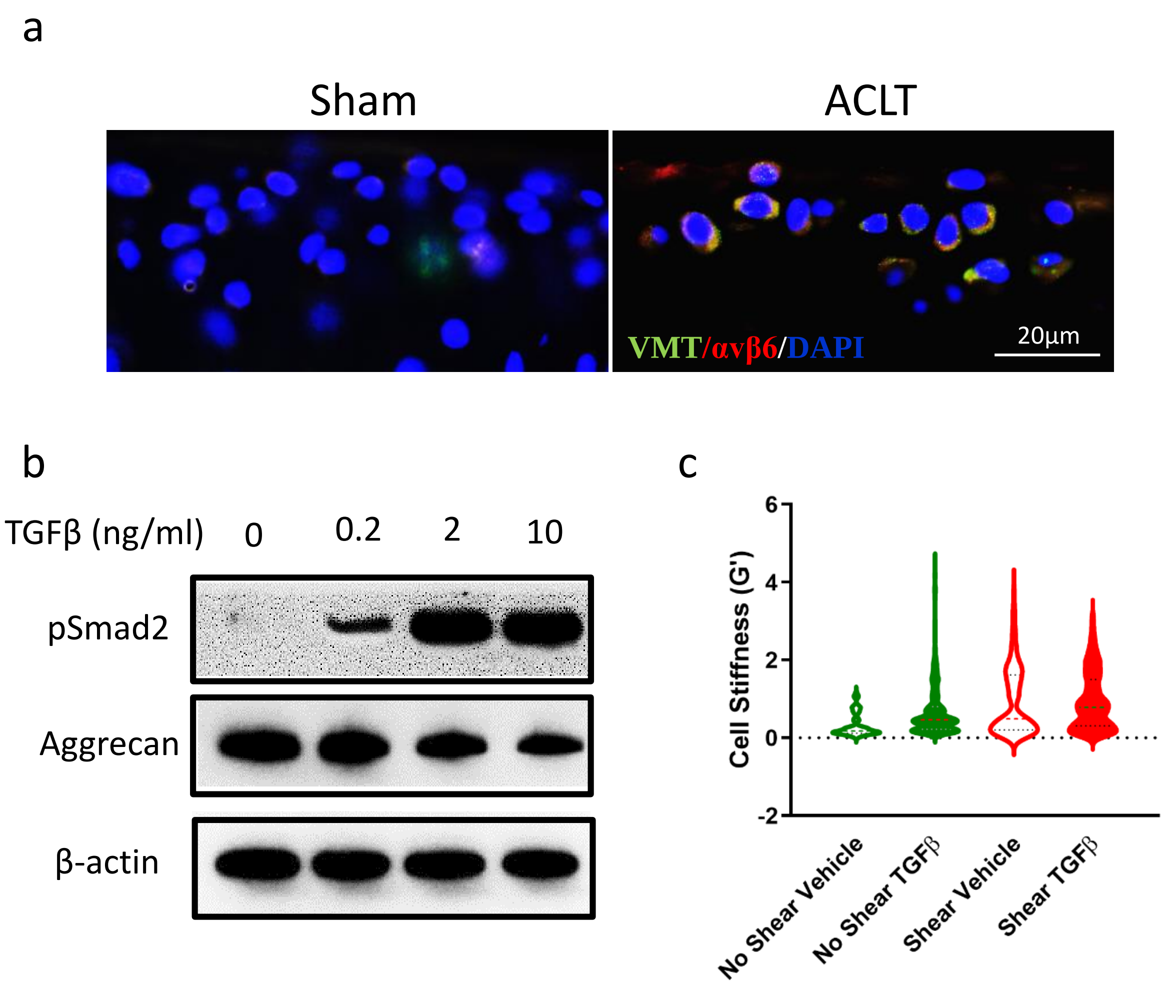

Supplementary Fig. 11 TGFβ increases chondrocyte stiffness. There is no dose dependent effect of TGFβ in inducing aggrecan secretion in chondrocytes. (a) Immunofluorescence staining of αVβ6 (red) and vimentin (VMT, green) in mouse articular cartilage that harvested at 1-month post-ACLT or sham operation. Nuclei were labeled with DAPI (blue). n=3 biological independent animals. (b) Primary chondrocytes that harvested from neonatal mice knee joint were incubated with TGFβ at different dosage. Incubating the cells with high levels of TGFβ did not further stimulate the aggrecan production by the chondrocytes. Experiments were repeated 3 times independently. (c) SV40 chondrocytes were subjected to shear stress (6.58 dynes/cm^2) or cultured in free well. These cells were treated with or without TGFβ (2ng/ml). The magnetic twisting cytometry was used to measure the cell stiffness. n=270-600 independent cells. Mean has been shown in dotted line.

SFig. 12

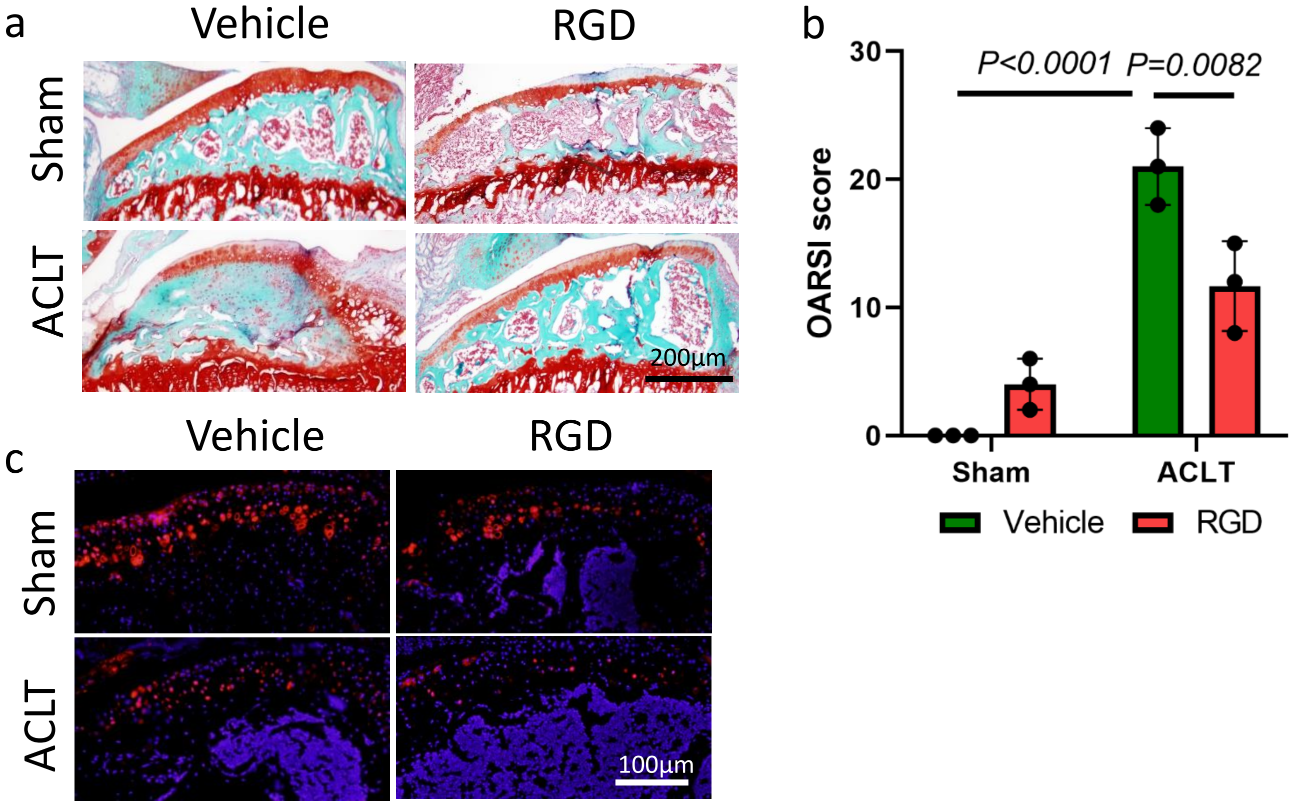

Supplementary Fig. 12 Intra-articular injection of RGD partially prevented ACLT induced cartilage degeneration. (a) Safranin-O staining of sagittal sections of mice knee joints. Mice were sacrificed at two months post ACLT or sham operation. Vehicle (PBS) or Arginylglycylaspartic acid (RGD) were injected intra-articularlly (5µM, 10µl, q.o.d). After ACLT, the cartilage and SB morphology were partially improved by RGD treatment relative vehicle treatment. Unfortunately, mild proteoglycan loss was also observed in RGD treatment sham mice possibly due to the global inhibition of all RGD binding domains in chondrocytes. (**b**) Quantitative analysis of OARSI Score base on the Safranin-O staining in (a). (**c**) Immunofluorescence staining of aggrecan (red) in sagittal sections of mice knee joints. Mice were intra-articularlly treated with vehicle or RGD after ACLT or sham operation. RGD treatment slightly reduced aggrecan production in both ACLT and sham operated mice. (**a-c**) n=3 biologically independent mice. Data are presented as mean values +/- SEM. Data was analyzed using one-way ANOVA LSD post hoc test.

SUPPLEMENTARY TABLE

Primer name	Sequence
Cre 5′	5’-CACTGCGGGCTCTACTTCAT
Cre 3′	5′−ACCAGCAGCACTTTTGGAAG −3′
lox1F	5′−GGTGACTCAATGTGTGACCTTCAGC−3′
lox1R	5′−CACAAATCAAGGATGACCAAACTGAG−3′

Supplementary table: The names and sequences of the primers used for Col2a-Cre^{ERT}: $\alpha V^{fl/fl}$ ($\alpha V^{-/-}$) mice genotyping. The primer pair of Cre 5’ and Cre 3’ were used to identify the Col2a-Cre^{ERT} while lox1F and lox1R were used to identify the $\alpha V^{fl/fl}$.
