# Supplementary material for: Mechanical stress determines the configuration of TGFβ activation in articular cartilage
Source: Nat Commun. 2021 Mar 17;12:1706. doi: 10.1038/s41467-021-21948-0 (PMC7969741; doi:10.1038/s41467-021-21948-0)
Supplement: Supplementary file 5 — Description of Additional Supplementary Files [file 41467_2021_21948_MOESM5_ESM.pdf]

Title: Supplementary Movie 1

Description: The transaxial and coronal view of micro-CT scan of the medial compartment tibias from 4 months old C57/BL6 mice one-month post-ACLT or sham surgery (left: ACLT, right: sham). The images were sectioned from proximal to distal in parallel.
